# Creating a provincial post COVID‐19 interdisciplinary clinical care network as a learning health system during the pandemic: Integrating clinical care and research

**DOI:** 10.1002/lrh2.10316

**Published:** 2022-05-15

**Authors:** Adeera Levin, Michelle Malbeuf, Alison M Hoens, Christopher Carlsten, Christopher J Ryerson, Alessandro Cau, Stirling Bryan, Jaclyn Robinson, Tamsin Tarling, Joanne Shum, Danielle C Lavallee

**Affiliations:** ^1^ Division of Nephrology University of British Columbia Vancouver British Columbia Canada; ^2^ Provincial Health Services Authority Vancouver British Columbia Canada; ^3^ Department of Medicine University of British Columbia Vancouver British Columbia Canada; ^4^ Providence Health Care Vancouver British Columbia Canada; ^5^ Michael Smith Health Research BC Vancouver British Columbia Canada; ^6^ Department of Physical Therapy University of British Columbia Vancouver British Columbia Canada; ^7^ Arthritis Research Canada Vancouver British Columbia Canada; ^8^ Centre for Clinical Epidemiology and Evaluation Vancouver Coastal Health Research Institute Vancouver British Columbia Canada; ^9^ Centre for Health Evaluation and Outcome Sciences St. Paul's Hospital Vancouver British Columbia Canada; ^10^ Centre for Heart Lung Innovation St. Paul's Hospital Vancouver British Columbia Canada; ^11^ Legacy for Airway Health Vancouver Coastal Health Research Institute Vancouver British Columbia Canada; ^12^ School of Population and Public Health University of British Columbia Vancouver British Columbia Canada; ^13^ Vancouver Coastal Health Authority Vancouver British Columbia Canada

**Keywords:** interdisciplinary care, knowledge translation, learning health system, post‐COVID‐19 care, provincial network

## Abstract

**Introduction:**

Coronavirus Disease‐2019 (COVID‐19) affects multiple organ systems in the acute phase and also has long‐term sequelae. Research on the long‐term impacts of COVID‐19 is limited. The Post COVID‐19 Interdisciplinary Clinical Care Network (PC‐ICCN), conceived in July 2020, is a provincially funded resource that is modelled as a Learning Health System (LHS), focused on those people with persistent symptoms post COVID‐19 infection.

**Methods:**

The PC‐ICCN emerged through collaboration among over 60 clinical specialists, researchers, patients, and health administrators. At the core of the network are the post COVID‐19 Recovery Clinics (PCRCs), which provide direct patient care that includes standardized testing and education at regular follow‐up intervals for a minimum of 12 months post enrolment. The PC‐ICCN patient registry captures data on all COVID‐19 patients with confirmed infection, by laboratory testing or epi‐linkage, who have been referred to one of five post COVID‐19 Recovery Clinics at the time of referral, with data stored in a fully encrypted Oracle‐based provincial database. The PC‐ICCN has centralized administrative and operational oversight, multi‐stakeholder governance, purpose built data collection supported through clinical operations geographically dispersed across the province, and research operations including data analytics.

**Results:**

To date, 5364 patients have been referred, with an increasing number and capacity of these clinics, and 2354 people have had at least one clinic visit. Since inception, the PC‐ICCN has received over 30 research proposal requests. This is aligned with the goal of creating infrastructure to support a wide variety of research to improve care and outcomes for patients experiencing long‐term symptoms following COVID‐19 infection.

**Conclusions:**

The PC‐ICCN is a first‐in‐kind initiative in British Columbia to enhance knowledge and understanding of the sequelae of COVID‐19 infection over time. This provincial initiative serves as a model for other national and international endeavors to enable care as research and research as care.

## 1 INTRODUCTION

The long‐term burden of Coronavirus Disease‐2019 (COVID‐19) infection on people and the health care system is unknown.^1^, ^2^, ^3^ International estimates indicate that 10% to 20% of individuals recovering from COVID‐19 experience long‐term complications.^4^ Given the multiplicity of organ systems affected and the susceptibility of individuals with substantial comorbidity to the infection, impacts are likely significant.^5^, ^6^, ^7^ As the focus of the pandemic shifts from acute management of disease, ensuring emphasis on treatment and support for patients with long‐term complications is needed.

The novelty and complexity of COVID‐19 combined with the evolving evidence around long‐term outcomes indicates the need to rapidly translate new knowledge that will inform the public, guide clinical practice, and drive health policy. To generate sufficient data to evaluate and advance patient care and health policy for COVID‐19, data from many patients across various geographic settings are needed. The variability in clinical presentation combined with small numbers of patients in any practice or jurisdiction necessitates broader collaborations as forums for learning. Learning Health Systems (LHS) provide a framework for organizing people and resources to address common challenges and support the rapid dissemination of findings to enable decision‐making.^8^, ^9^, ^10^ Distinguishing characteristics of a LHS include recognizing the need to learn from routine patient care and the enablement to do so through fostering a culture of inquiry, facilitating partnerships between research and clinical practice, and purpose‐built robust data infrastructure.^8^, ^10^, ^11^, ^12^, ^13^

British Columbia (BC), Canada's westernmost province, announced its first COVID‐19 case in January 2020 and progressed to a formal public health emergency 2 months later as community transmission increased.^14^, ^15^ BC's landmass spans approximately 950 000 km^2^ with an estimated population of 5 million people. At the pandemic's start, BC experienced variable incidences of the virus and uneven access to healthcare services across the geographically diverse province, including access to testing, acute care, and dedicated outpatient follow‐up. The post COVID‐19 Interdisciplinary Clinical Care Network (PC‐ICCN) was conceived in May 2020 in recognition of an emerging need for specialized coordinated care for a subset of post COVID‐19 patients with persistent symptoms (now recognized as long‐haul COVID, long COVID, or Post Acute Sequelae of COVID‐19, PASC).^16^ Modelled as a LHS, the PC‐ICCN evolved iteratively to address patient care goals, facilitate clinical collaboration, and enhance research integration.^17^, ^18^ Herein, we describe the PC‐ICCN and data infrastructure developed to support ongoing clinical care and research.

## 2 PC‐ICCN OVERVIEW AND NETWORK OBJECTIVES

Based on the known physiology of the whole‐body distribution of the angiotensin converting enzyme‐2 receptor and the virus binding to the receptor, the possibility of multisystem involvement became apparent early in the pandemic^19^, ^20^ fostering an urgent need for interdisciplinary collaboration. The PC‐ICCN emerged through collaboration among over 60 clinical specialists, researchers, patients, and health administrators.

The PC‐ICCN aims to identify, implement, and evaluate clinical care programs and health policy initiatives that improve the care of patients with persistent symptoms post COVID‐19 using a LHS approach; to ensure rapid knowledge translation of initiatives to key stakeholders, including patients and physicians; and, to embed research infrastructure within clinical care to facilitate learning and evidence generation to support patient care, advance clinical guideline development, and support healthcare policy. Uniquely the network was created in the context of the unfolding pandemic, without dedicated funding, no single source of health care information, and challenges in outpatient care delivery at different times in different physical locations.

The primary goals of the network were the following: (a) to demonstrate the ability to stand up interdisciplinary clinics offering care and education to patients suffering from long COVID, with standardized follow up and data capture in order to (b) acquire the expertise and experience to deliver high quality interdisciplinary care, (c) to develop accessible tools for primary care physicians and patients to enable capacity building with that acquired expertise, and (d) to use data acquired in this context to inform the evolution of care models. The secondary goals were to normalize the embedding of research into clinical care, and to create a culture of collaboration across different clinical and research teams.

## 3 THE PC‐ICCN AS A LEARNING HEALTH SYSTEM

**FIGURE 1:**
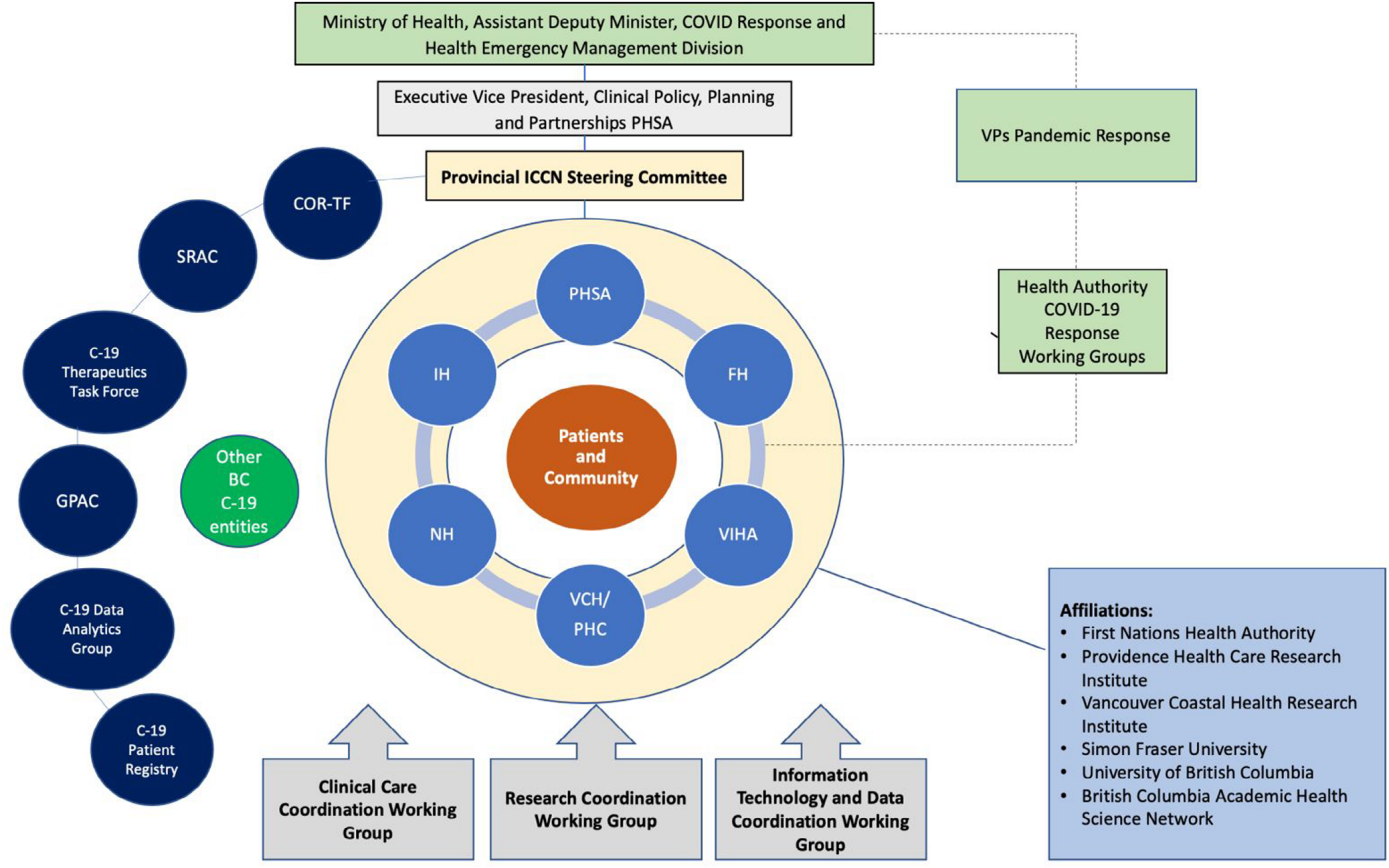
The structure of the Network and Registry within the province of BC. SRAC, Strategic Research Advisory Council^21^; GPAC, Guidelines and Protocols Advisory Committee^22^; COR‐TF, Clinical Operations and Research Task Force^23^; PHSA, Provincial Health Services Authority; FH, Fraser Health; VIHA, Vancouver Island Health Authority; VCH, Vancouver Coastal Health; PHC, Providence Healthcare; NH, Northern Health; IH, Island Health

LHSs represent environments wherein culture both supports and facilitates continuous improvement in patient care and care processes through fostering community, providing infrastructure, and aligning clinical, quality, and academic incentives. Menear et al propose six pillars as the foundation of a LHS: scientific, social, technological, policy, legal and ethical.^10^ The components of the PC‐ICCN aligned with these pillars, presented and defined in Table 1, serve to enable communities to complete learning cycles around prioritized issues.^10^ The PC‐ICCN aligns with the LHS framework by leveraging data generated as part of clinical care to create evidence needed to address questions patients, clinicians, and policy‐makers need answers to in selecting or providing patient care. The complete structure of the PC‐ICCN Network is depicted in Figure 1. Key enabling features of the PC‐ICCN include provision of specialized coordinated patient care with systematic data collection; alignment between clinical needs and health services research; and, education and knowledge translation around post COVID‐19 recovery care to support PC‐ICCN stakeholders inclusive of patients.

**TABLE 1:** PC‐ICCN Alignment with LHS Pillars

Pillar^a^	Pillar description^a^	PC‐ICCN components
Scientific	Encompasses scientific infrastructure, resources, and capacity building programs that support knowledge generation and translation. Reflected in the scientific pillar is recognition of diverse methodologies relevant to real‐world contexts. This includes quality improvement, system science, data science, implementation science, and patient‐oriented research methodologies.	PC‐ICCN leadership comprising clinical, health systems, and patient‐oriented research expertise. PC‐ICCN patient registry available to support clinical outcomes and health services research in this population. Portal to support scientific infrastructure via partnership for research http://www.phsa.ca/our‐services/programs‐services/post‐covid‐19‐recovery‐clinics. Research scholars supported for PC ICCN work. Research assistants, statisticians, and methodologists dedicated to PC ICCN.
Social	Aims to breakdown silos and create community around learning. Reflected in the social pillar are individuals and networks interacting with and within the health system. This includes patients, family members, care partners, community members, clinicians, health care team members, administrators, policymakers, researchers, industry partners or other experts.	Working groups established within each regional health authority inclusive of patients, clinicians, professional services, and researchers. The development of educational materials and standardization of care pathways was made possible through working groups funded through Doctors of BC Shared Care and Health System Redesign initiatives. Established linkages with existing services (ie, RACE line and RTVS) enable comprehensive care. Clinical community development through PCRC multidisciplinary rounds. Multidisciplinary clinics with telehealth/ virtual outreach to all geographic regions in the province. Education and knowledge translation to physicians, patients and other stakeholders available on a central website: http://www.phsa.ca/our‐services/programs‐services/post‐covid‐19‐recovery‐clinics
Technological	Enables learning through IT infrastructure, systems, and resources embedded in care that allow data to be captured, aggregated, analyzed, and acted upon by decision‐makers.	Patient registry for all consenting patients enrolled in the ICCN providing systematic data collection. Biobank capabilities allow for data collection necessary to support the network's goals. Patient registry linkage with other administrative data support a prospective database of patients
Policy	Promotes governance structures, clear policies, adequate financing, and clear accountability measures. Reflected in the policy pillar are established processes for decision‐making, roles and responsibilities, and metrics for the Learning Health Systems to facilitate transparency and accountability for work conducted.	Provincial steering committee comprising of clinicians, leaders from all health authorities, the Ministry of Health, patient partners, and researchers to increase the accountability of the PC‐ICCN with bi‐directional support from the committee to help address roadblocks experience. Governance structures including a core steering group, a clinical care coordination working group, a research coordination working group, and a data information coordination working group.
Legal	Provides guidance and scope to the conduct of the LHS within regulatory and legal realm. This encompasses privacy legislation for safeguarding personal health information as well as regulatory guidance for professional practice and healthcare delivery.	PROMIS is an administrative health care database that stores fully identified patient‐level data and is managed by BC Provincial Renal Agency under regulation by the provincial government of BC.
Ethical	Promotes structures and processes aimed to guide ethical approach to continuous learning. Embedded learning in health systems may create a lack of clear distinction between clinical practice, quality improvement, evaluation, and research.	All research applications must have a separate Research Ethics Board approval prior to PC‐ICCN data release to a requesting researcher. The PC‐ICCN Biobank has approval from the University of British Columbia Clinical Research Ethics Board

^a^ Adapted from Reference 10.

The PC‐ICCN has evolved and expanded according to the changing burden of COVID‐19 case loads in BC, resource availability, and increasing knowledge of the condition.^24^ The PC‐ICCN is responsible for provincial centralized triage, clinical coordination, research integration, and accountability for outcomes. Leveraging BC's existing health services and data infrastructure, PC‐ICCN created a formal patient registry within a current provincial information system; established working groups to advance clinical care, education, and research; and established a provincial steering committee comprising patients, physicians, researchers, nurses, administrators, and analysts. A provincial website with patient‐ and physician‐facing materials was developed and launched with a detailed communication plan to facilitate awareness and uptake. A provincially accessible accredited education program and a provincial physician “help‐line” (both email and telephone‐based) were established to support physicians. Finally, research scholars and analytic staff have been hired to support a robust set of outputs to inform care and identify research questions.

Patient engagement occurred at the outset of the PC‐ICCN development and has evolved over time. Patient and public engagement is essential in all research. However, in the context of a pandemic, where rapid development of evidence is needed, a patient‐oriented approach is critical to support the rapid growth and translation of research.^25^, ^26^ During the nascent stage of the network development, experienced patient research partners from established kidney and lung research networks (inclusive of research registries) and patients post COVID‐19 who were new to patient engagement in research, were welcomed to ensure a patient‐oriented approach in network structure, governance, and subcommittee work.^27^, ^28^ These patient partners grounded efforts on the lived experience. Given the elevated risk for significant complications from COVID‐19, patient partners provided a welcomed perspective on concerns and questions relevant for members of the patient community and the greater public. Best practices in patient engagement, including financial compensation for participation, were incorporated.^29^

## 4 INTEGRATING CARE AND RESEARCH

### 4.1 Post COVID‐19 recovery care

**FIGURE 2:**
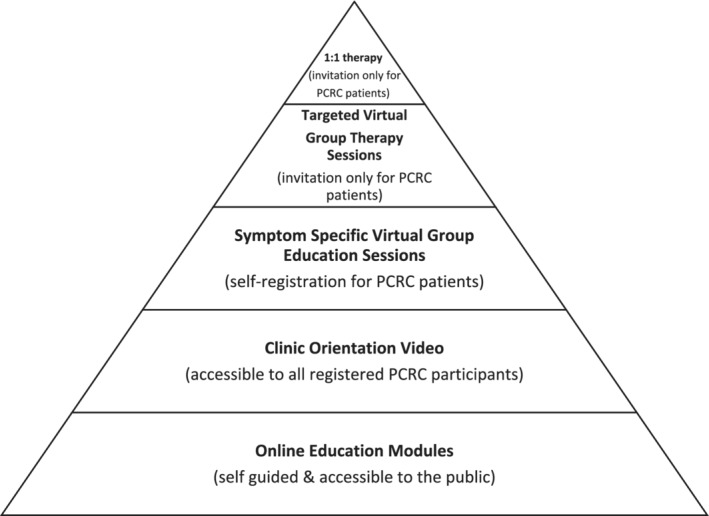
Patient access to Allied Health facilitated education and therapy

At the core of the PC‐ICCN are five Post COVID‐19 Recovery Clinics (PCRCs), which provide direct patient care that includes standardized testing and education at initially regular follow‐up intervals for a minimum of 12 months post enrolment. The PCRCs are available to all patients across BC who meet clinical eligibility criteria with referral by their primary care provider. Both in‐person and virtual health options are made available. PCRCs are interdisciplinary, integrated with primary care providers, and co‐designed by survivors of COVID‐19. Patients attending clinics undergo routine medical testing and receive education and resources to support their recovery. Facilitated education and therapy tailored to the needs and preferences of patients is provided (Figure 2) and range from web‐based modules to 1:1 therapy sessions for enrolled PCRC patients.

### 4.2 PC‐ICCN patient registry

Patient registries embedded into care delivery support the standardization of data collection and enhance clinician collaboration resulting in improved patient care.^30^, ^31^, ^32^ In addition, such registries advance the understanding of the natural history and progression of disease and thus, the ability to optimize care for current and future patients.^33^ Initiated in July 2020, the PC‐ICCN patient registry captures data on all referred patients to a PCRC at the time of referral, with data stored in a fully encrypted Oracle‐based database. The database was adapted from an existing provincial database for kidney diseases and solid organ transplant recipients, has a separate module to include PCRC patients, is accessible only to those care providers, and leverages the existing expertise, linkages, and structure of the existing provincial database. Eligible patients for the registry are those with confirmed COVID‐19 infection by laboratory testing or epi‐linkage who were either hospitalized with COVID‐19 infection or who were managed as outpatients with symptoms persisting for 12 weeks or more that cannot be explained by an alternative diagnosis. This is consistent with the World Health Organization definition of Long COVID.^16^

**FIGURE 3:**
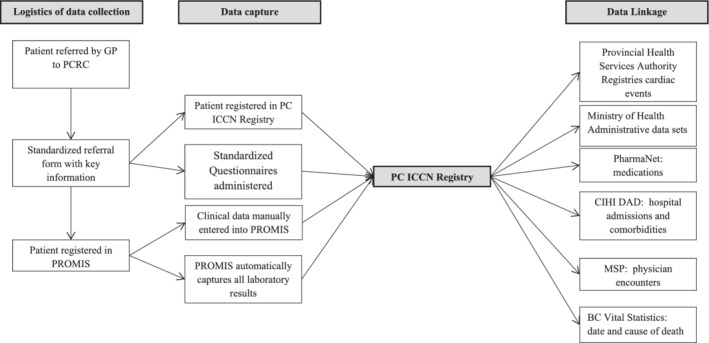
The logistics of data capture for the Registry. PC‐ICCN, Post COVID‐19 Interdisciplinary Clinical Care Network; PROMIS, Patient Registration and Outcomes Management Information Systems; CIHI DAD, Canadian Institute of Health Information Discharge Abstract Database; MSP, Medical Services Plan

A core dataset inclusive of clinical, patient‐reported, and laboratory data (Table 2) is captured on all patients referred to the PCRC as detailed in Figure 3. Once a patient is registered in the registry, laboratory results are automatically uploaded through established and sanctioned interfaces. Patients are assessed using standardized testing protocols, including symptom questionnaires vetted or developed by a provincial working group of patients and interdisciplinary clinicians. Where validated questionnaires for symptoms of fatigue, breathlessness, depression existed, these were used. The core dataset (Table 2) includes demographic, clinical, laboratory, and imaging data, with a balance between patient burden and comprehensiveness.

**TABLE 2:** Core outcomes assessment schedule

Item	3 months (Baseline)	6 months	12 months	18 months
Patient reported				
Date of birth	X			
Sex	X			
Date of symptom onset	X			
Smoking history	X			
Cough severity^a^	X	X	X	X
Shortness of breath^b^	X	X	X	X
Quality of life^c^	X	X	X	X
Psychiatry screen^d^	X	X	X	X
Traumatic events^e^	X	X	X	X
Fatigue severity scale^f^	X	X	X	X
Clinical tests				
30 second sit to stand^g^	X		X	
Height	X			
Weight	X	X	X	X
Blood pressure	X	X	X	X
Laboratory^h^				
CBC, diff	X	X	X	X
Albumin	X	X	X	X
Electrolytes, creatinine	X	X	X	X
LFTs	X	^i^	^i^	^i^
NT pro‐BNP	X	^i^	^i^	^i^
Troponin	X	^i^	^i^	^i^
CRP	X	X	X	X
D‐Dimer, Fibrinogen^j^	X	X	X	X
Ferritin	X	X	X	X
LDH	X	^i^	^i^	^i^
Urine ACR	X	X	X	X
Urine analysis	X	^i^	^i^	^i^
Urine microscopy	X	^i^	^i^	^i^
Diagnostics (hospitalized patients only)				
Chest CT^j^	X		^i^	
Echo^j^	X		^i^	
PFTs and 6MWT^j^	X		^i^	

^a^ Cough Visual Analogue Scale (VAS).^34^

^b^ The University of California, San Diego Shortness of Breath Questionnaire (UCSD SOBQ).^35^

^c^ EQ‐5D‐5L.^36^

^d^ Generalized Anxiety Disorder‐2 (GAD‐2)^37^; Patient Health Questionnaire‐2 (PHQ‐2)^38^; CAGE Adapted to Include Drugs.^39^

^e^ Primary Care PTSD Screen for DSM‐5 (PC‐PTSD‐5).^40^

^f^ Fatigue Severity Scale (FSS).^41^

^g^ 30 second sit to stand.^42^

^h^ Laboratory data protocol reflected for hospitalized patients only.

^i^ Only order this test if previously had an abnormal result.

^j^ Removed from protocol Fall 2021.

### 4.3 PC‐ICCN biobank

All patients referred to a PCRC are invited to participate in the provincial biobank network, BC Covid Biobank Network (BCCBN). The PC‐ICCN is a registered biobank under the Canadian Tissue Repository Network program, complies with guidance for quality biobanking, and was created in accordance with international standards with the goal of improving health outcomes under the umbrella of the BCCBN. The PC‐ICCN biobank collects blood samples from consenting patients, processes them in a standardized manner, and generates high quality derivatives for downstream research. Samples may be linked to the clinical information in the PC‐ICCN Patient Registry and other data sets. Annotated samples can then be accessed by researchers with approval from the PC‐ICCN review committee and appropriate research ethics boards as warranted.

The core dataset is based on standardized protocols and visit intervals. This integrates high quality data capture as part of clinical practice allowing for data aggregation and analyzes for the purpose of generating new knowledge, to address the existing void of evidence‐based treatment recommendations for post COVID‐19. Further, data can be linked to other governmental administrative databases including: outpatient medication dispensations; the Canadian Institute of Health Information Discharge Abstract Database that records hospital admissions, discharge diagnoses, and comorbidities; the Medical Services Plan which identifies all visits to physicians; and Vital Statistics Database that registers causes and date of death, and the Provincial Immunization Registry. This integration of granular clinical information with administrative data sets is intended to provide a rich source by which to support rapid learning cycles aimed to answer questions of interest to patients, clinicians, researchers, and policy makers.

## 5 ACCESS TO DATA AND PROCESSES FOR INCORPORATION OF FINDINGS INTO CLINICAL CARE

Data access for researchers is governed by the policies and procedures dictated by the academic institutions and Ministry of Health. Proposals are submitted through a harmonized ethics process. Dedicated statisticians and analysts with access to comprehensive data sets and linkage processes work with researchers to answer questions.^43^ Specific questions regarding utility of processes and testing strategies are subject to the same process though expedited, given the real time need for the data to inform care. Feedback and review of data to clinical care teams is done through regular bi‐weekly meetings. Decision making for changes are based on consensus after review of data, and communicated through various established groups of operational managers and clinical teams. Research assistants, patients, clinicians and researchers are invited to regular research meetings as well as to more formal education sessions. All meetings to date have been conducted virtually (see below for examples of changes to clinical protocols based on this process).

## 6 KNOWLEDGE TRANSLATION FOR PRACTICE AND POLICY

A core function of the PC‐ICCN is to facilitate education and knowledge translation with and to physicians, patients, and other stakeholders. Content development is led by a working group comprising patients, clinicians, and knowledge translation experts. A multimodal approach to education and training (ie, written materials, videos, webinars, PowerPoint presentations, infographics) is used recognizing the different ways in which information is preferably accessed and used by various stakeholder groups. Educational rounds occur on a monthly basis and support clinical learning and knowledge exchange with community practitioners. Based on the Project ECHO (Extension of Community Health Outcomes) model, these one‐hour virtual events use case‐based learning around a community‐identified topic to improve care for patients experiencing symptoms post COVID‐19.^44^, ^45^ Patient information including information sheets, tools for tracking and managing post COVID‐19 symptoms, and links to COVID‐19 resources are available through the PC‐ICCN website. Where possible, information is made available in multiple languages including Punjabi, Tagalog, and Chinese. All materials are freely accessible and routinely updated.

## 7 DISCUSSION

The PC‐ICCN and its PCRCs represent a LHS approach for integrating prospective data collection from COVID‐19 patients into a provincial clinical care program. The data enable the development and evaluation of health policy and health services aimed at improving outcomes. Further, the involvement of and collaboration among patients, clinicians, administrators, policy‐makers, and researchers enhances the ability to rapidly disseminate and mobilize new knowledge.

Before PC‐ICCN, as is the current state in many national and international jurisdictions, there was no consistent formalized framework or data platform to inform the care of these patients. As of April 2022, BC has had over 356 000 cases of COVID‐19. Of total cases, 19 897 have been hospitalized, and 3004 have died.^46^ Within BC, 35 364 people have been referred to one of the now five existing PCRCs. Compared to the total number of post COVID‐19 patients, this low number of referrals reflects the delays in establishing PCRCs and the time it takes to build capacity within new clinics. To date, 5364 patients have been referred, with an increasing number and capacity of these clinics, and 2354 people have had at least one clinic visit. A mixed methods approach has been conducted to evaluate patient experiences in the clinic (using surveys and focus groups), as have formal evaluations of educational events for care providers, as part of the “fabric” of the network. Publications describing the results of these efforts, as well as baseline cohort descriptions of the distribution of symptom complexes, common and specialized laboratory tests, the relationship between these, and changes over time are in preparation.

The integration of data capture within the larger framework of health administration allows for rapid learning. Since the inception of routine data capture, clinicians with support from data analysts, have analyzed diagnostic and laboratory data to understand correlation with patient outcomes. Examples of key “changes” include the removal of routine capture of diagnostic data including chest computed tomography, echocardiogram, pulmonary function tests, and six‐minute walking tests following an evaluation of the utility for clinical decision‐making. This change reduced the burden of testing on patients and the health care system. In addition, the panel of routine “surveillance bloodwork” has undergone similar refinement. Initially, D‐dimer, a laboratory test used as predictor for severity of COVID‐19 infection, was included in the routine bloodwork panel, on the basis that it may help with surveillance and follow up. However, further detailed analysis with clinicians and statisticians led to the recognition that the test was of limited value for patient care, had the potential to lead to additional investigations and was a burden to patients and clinical workflow. Thus, through a consensus process, the decision was made to remove the lab value from the protocol. The patient registry will continue to allow for robust evaluation of clinical care programs developed by the PC‐ICCN.

This model has proven effective at improving the outcomes of chronic conditions in BC, for example demonstrating that patients with chronic kidney disease managed in interdisciplinary care clinics have better metabolic parameters and improved survival on dialysis compared to those managed solely by nephrologists.^47^ We anticipate being able to describe similar findings for the PC‐ICCN. Combining the patient registry with health service delivery has the additional benefit of improving the scope and reducing the cost of data capture for this new condition. Ongoing evaluation of referral patterns as well as visits and discharges from the clinic during this unique, evolving clinical context amidst a pandemic are critical to further information models of care and resource needs.

Many other COVID‐19 initiatives have either been specialist clinics (eg, respiratory, psychiatry), single visits, or focused exclusively on collecting research data.^48^ The PC‐ICCN is uniquely built on existing provincial health delivery infrastructure, links laboratory and administrative databases to minimize laborious data entry, improves data quality and quantity, and is predicated on a longitudinal follow‐up model to care for and study the long‐term sequelae of COVID‐19. In addition, this approach allows for a complete capture of relevant patient information to support patients who live across the large and geographically diverse province of BC. In studying novel conditions such as post‐acute sequelae of COVID‐19, the comprehensive data capture of a large number of patients over extended periods is essential. We recognize that not all patients with long‐term sequelae were referred to the network clinics, thus our sample of patients represent a subset of all those suffering from the condition. Through ongoing collaborations with other jurisdictions, and researchers, we will compare and contrast those referred with those who were not, creating a rich dataset for gaining insights into resource utilization, testing patterns, and symptom burdens over time. The dynamic and ever changing circumstances of the pandemic (eg, no vaccination available, partial then complete vaccination schedule, changing variant exposures, and changing knowledge base) makes this work particularly challenging.

There exist other large‐scale research networks like the PC‐ICCN that were developed to inform the ongoing effort against Long COVID. The National Institutes of Health‐funded Researching COVID to Enhance Recovery (RECOVER) consortium is a nationwide network of researchers, scientists, clinicians and patients whose objective is to better understand Long COVID and identify novel treatment options for individuals living with this condition.^49^ Coordination of the research activities of this extensive network is achieved by four RECOVER “cores,” or groups of investigators, in clinical science, data science, administration and biorepository management. In addition, a number of evidence synthesis groups have been developed both nationally and internationally since the inception of the network.^50^, ^51^ These sources of information are used in the preparation of the various rounds, educational sessions and clinical meetings that are part of the network functioning. The PC‐ICCN is anticipated to produce a rich dataset with clinically relevant outcomes that support research in this evolving area. Since inception, the PC‐ICCN has received over 30 research proposal requests, for access to data, access to bio samples, and requests for collaboration. This is aligned with the goal of creating infrastructure to support a wide variety of research relevant to post COVID‐19. Canada's universal health care system helps reduce some of the bias that results from differential access to care. However, there remains differential incidence of COVID‐19 around the province and variable access to healthcare. Having a provincial database allows inclusion of any patient, independent of their location, thereby improving generalizability and strengthening clinical outcomes and health services research. Furthermore, the coordinated provincial strategy for identifying and providing long‐term follow‐up for patients with post COVID‐19 sequelae will facilitate recruitment into prospective clinical trials and other multicenter studies. The process of vetting and prioritizing the research proposals is in evolution, as data completeness, relevance, and the aforementioned changing landscape challenge us.

There are potential threats to the sustainability of the PC‐ICCN. The initial development occurred through the intentional though volunteer effort of clinicians, researchers, and health system administrators to organize care such that knowledge could more easily be generated in a way that rapidly informed patient care, at a time where little was known. Through persistent effort, PC‐ICCN leadership secured timebound funding and dedicated support through research and health system funding mechanisms. Long‐term sustainability of the PCRCs and broader network are unknown at this time, and the rapidly changing face of the pandemic and its consequences further challenge the health care systems ability to adapt. There are several limitations to the data collected in the patient registry. First, in an attempt to improve the feasibility of data collection and the geographic distribution of patient enrolment, decisions were made regarding the need to collect a limited set of core clinical variables vs more expansive data capture reflective of research‐driven interests. Not all imaging tests are uploaded to the registry, but can be accessed from clinical records as needed, and thus some research questions will require review of individual patient records. Furthermore, the patient registry only enrolls patients referred to the clinic and will therefore not be generalizable to all patients who have had COVID‐19, and particularly those who have symptoms not deemed candidates for referral by their general practitioner. Plans exist to expand the database to capture all patients with persistent symptoms through different mechanisms, including self‐referral for registry purposes only; however, this requires changes to common practice and workflow not yet resourced. Finally, there is a need for regular data validation methods to ensure correct classification and data entry. Pragmatic and robust data validation processes are being developed and will be the subject of a future publication once a sufficient sample of patient records is available.

## 8 CONCLUSION

The COVID‐19 pandemic prompted development of the PC‐ICCN with a learning health system framework in which patient care is provided while advancing new knowledge in real‐time. PC‐ICCN represents a pan‐provincial integrated system to understand and improve the care of patients who have persistent symptoms post COVID‐19 infection. The uniqueness of this network is based on having virtual and in person encounters; education for patients, providers and policy makers; access to research quality data; and, collaboration across health, research, and policy systems. The future of the pandemic and long‐term impacts on individuals and systems remains unknown. This structure permits ongoing learning to inform care and policy, while serving as a model for future novel health threats. We would suggest that ultimately the “success” of the network would include the acquisition and dissemination of knowledge to enable primary care providers to manage patients with persistent symptoms, the normalization of research approaches into clinical care, the generation of collaborations across different specialities for research and care purposes, and the evolution of the care model, based on data, to meet the changing needs of patients and health care providers in the context of an evolving clinical condition. We are working towards this.

## AUTHOR CONTRIBUTIONS

All authors (Adeera Levin, Michelle Malbeuf, Alison M Hoens, Christopher Carlsten, Christopher J Ryerson, Alessandro Cau, Stirling Bryan, Jaclyn Robinson, Tamsin Tarling, Joanne Shum, Danielle C Lavallee) have been involved in developing and implementing the PC‐ICCN, including the logistics and processes around patient registration and data capture. In addition, all authors contributed to manuscript writing, editing and submission.

## CONFLICT OF INTEREST

The authors declare no conflicts of interest.

## ETHICS STATEMENT

PC‐ICCN data is maintained in PROMIS, which is under regulation by governmental agencies in BC (the BC Renal and the Provincial Health Services Authority). Anyone interested in accessing PROMIS data for non‐commercial research purposes must complete a data application request (which will be vetted by the PC‐ICCN) and secure ethics approval through the University of British Columbia. Linkage of PROMIS data to other administrative databases may require additional approval from appropriate data stewards.
